# Antirotavirus IgA seroconversion rates in children who receive concomitant oral poliovirus vaccine: A secondary, pooled analysis of Phase II and III trial data from 33 countries

**DOI:** 10.1371/journal.pmed.1003005

**Published:** 2019-12-30

**Authors:** Julia M. Baker, Jacqueline E. Tate, Juan Leon, Michael J. Haber, Benjamin A. Lopman

**Affiliations:** 1 Department of Epidemiology, Rollins School of Public Health, Emory University, Atlanta, Georgia, United States of America; 2 Division of Viral Diseases, National Center for Immunization and Respiratory Diseases, Centers for Disease Control and Prevention, Atlanta, Georgia, United States of America; 3 Hubert Department of Global Health, Rollins School of Public Health, Emory University, Atlanta, Georgia, United States of America; 4 Department of Biostatistics and Bioinformatics, Rollins School of Public Health, Emory University, Atlanta, Georgia, United States of America; The University of Melbourne School of Historical and Philosophical Studies, AUSTRALIA

## Abstract

**Background:**

Despite the success of rotavirus vaccines over the last decade, rotavirus remains a leading cause of severe diarrheal disease among young children. Further progress in reducing the burden of disease is inhibited, in part, by vaccine underperformance in certain settings. Early trials suggested that oral poliovirus vaccine (OPV), when administered concomitantly with rotavirus vaccine, reduces rotavirus seroconversion rates after the first rotavirus dose with modest or nonsignificant interference after completion of the full rotavirus vaccine course. Our study aimed to identify a range of individual-level characteristics, including concomitant receipt of OPV, that affect rotavirus vaccine immunogenicity in high- and low-child-mortality settings, controlling for individual- and country-level factors. Our central hypothesis was that OPV administered concomitantly with rotavirus vaccine reduced rotavirus vaccine immunogenicity.

**Methods and findings:**

Pooled, individual-level data from GlaxoSmithKline’s Phase II and III clinical trials of the monovalent rotavirus vaccine (RV1), Rotarix, were analyzed, including 7,280 vaccinated infants (5–17 weeks of age at first vaccine dose) from 22 trials and 33 countries/territories (5 countries/territories with high, 13 with moderately low, and 15 with very low child mortality). Two standard markers for immune response were examined including antirotavirus immunoglobulin A (IgA) seroconversion (defined as the appearance of serum antirotavirus IgA antibodies in subjects initially seronegative) and serum antirotavirus IgA titer, both collected approximately 4–12 weeks after administration of the last rotavirus vaccine dose. Mixed-effect logistic regression and mixed-effect linear regression of log-transformed data were used to identify individual- and country-level predictors of seroconversion (dichotomous) and antibody titer (continuous), respectively. Infants in high-child-mortality settings had lower odds of seroconverting compared with infants in low-child-mortality settings (odds ratio [OR] = 0.48, 95% confidence interval [CI] 0.43–0.53, *p* < 0.001). Similarly, among those who seroconverted, infants in high-child-mortality settings had lower IgA titers compared with infants in low-child-mortality settings (mean difference [β] = 0.83, 95% CI 0.77–0.90, *p* < 0.001). Infants who received OPV concomitantly with both their first and their second doses of rotavirus vaccine had 0.63 times the odds of seroconverting (OR = 0.63, 95% CI 0.47–0.84, *p* = 0.002) compared with infants who received OPV but not concomitantly with either dose. In contrast, among infants who seroconverted, OPV concomitantly administered with both the first and second rotavirus vaccine doses was found to be positively associated with antirotavirus IgA titer (β = 1.28, 95% CI 1.07–1.53, *p* = 0.009). Our findings may have some limitations in terms of generalizability to routine use of rotavirus vaccine because the analysis was limited to healthy infants receiving RV1 in clinical trial settings.

**Conclusions:**

Our findings suggest that OPV given concomitantly with RV1 was a substantial contributor to reduced antirotavirus IgA seroconversion, and this interference was apparent after the second vaccine dose of RV1, as with the original clinical trials that our reanalysis is based on. However, our findings do suggest that the forthcoming withdrawal of OPV from the infant immunization schedule globally has the potential to improve RV1 performance.

## Abstract 2

In a secondary, pooled analysis of phase II and III trial data from 33 countries, Julia M Baker and colleagues investigate anti-rotavirus IgA seroconversion rates in children who receive concomitant oral poliovirus vaccine.

## Author summary

**Why was this study done?:**

- Rotavirus vaccination is the primary method for preventing rotavirus infections and reducing the severity of rotavirus gastroenteritis among young children globally.
- Rotavirus vaccine underperformance (lower vaccine efficacy/effectiveness and immunogenicity) among children in high-child-mortality settings where the incidence of severe disease and death is highest hinders further progress in reducing the rotavirus burden.
- Infant characteristics, such as malnutrition, age at first rotavirus dose, and concomitant receipt of oral poliovirus vaccine (OPV), may impact rotavirus vaccine immunogenicity.
- Identifying individual-level factors that impact vaccine immunogenicity is critical for determining modifiable vaccine strategies or interventions for enhancing vaccine performance.

**What did the researchers do and find?:**

- We analyzed pooled, individual-level data on 7,280 vaccinated infants from 22 GlaxoSmithKline Phase II and III clinical trials of the monovalent rotavirus vaccine (RV1) across 33 high- and low-child-mortality countries/territories.
- We examined the relationship between individual- and country-level predictors and immune response to RV1 vaccination measured via serum antirotavirus immunoglobulin A (IgA) seroconversion and serum antirotavirus IgA titer.
- Our findings suggest that OPV given concomitantly with the first and second doses of rotavirus vaccine reduces antirotavirus IgA seroconversion.
- We did not find the same modifiable characteristics associated with seroconversion to be associated with postvaccine antirotavirus IgA titers among infants who seroconverted.

**What do these findings mean?:**

- Factors that may predict whether an infant responds to RV1 (seroconversion) may not also predict the intensity of the response (titer) given seroconversion.
- Our findings highlight the importance of concomitant OPV and RV1 administration and provide encouraging evidence to suggest that the eventual withdrawal of OPV from national immunization programs could improve rotavirus vaccine performance.

## Introduction

Globally, rotavirus is the leading cause of severe diarrheal disease among infants and children under 5 years of age, estimated to cause 128,500–215,000 deaths in this age group each year during 2013–2016, when global vaccination coverage was <25% [1,2]. The virus is highly infectious, and improvements in water, sanitation, and hygiene conditions have limited impact in reducing its spread [3]. Rotavirus is a ubiquitous infection among young children [1], the majority of whom will experience at least one rotavirus infection in the first 2 years of life [4–6]. As such, vaccination is an essential public health measure for preventing infections and reducing the severity of rotavirus gastroenteritis [7].

Currently, four live attenuated, oral rotavirus vaccines administered during infancy have received World Health Organization (WHO) prequalification: GlaxoSmithKline’s (GSK) monovalent rotavirus vaccine (Rotarix, “RV1”), Merck’s pentavalent vaccine (RotaTeq), the Serum Institute of India’s pentavalent vaccine (Rotasiil), and Bharat Biotech’s monovalent vaccine (Rotavac) [8]. Since the pivotal clinical trial results were released in 2006 [9–11], rotavirus vaccines have been integrated into the national immunization programs of approximately 96 countries [12]. The introduction of these vaccines has led to dramatic reductions in rotavirus disease in many settings [9,10,13–15]. Despite this success, rotavirus remains a leading cause of severe diarrheal disease among young children [2] and continues to be the predominant cause of hospitalization for severe diarrheal disease, even in some countries where the vaccine is in widespread use [16,17].

A primary obstacle preventing further reductions in the rotavirus burden is vaccine underperformance among children in settings where the incidence of severe disease and death is highest [2,18]. An estimated 85% of rotavirus-related deaths occur among children in Africa and Asia [2], and in these high-child-mortality settings, vaccine effectiveness against severe gastroenteritis is approximately 50%–60% [19]. In contrast, vaccine efficacy is greater than 90% in low-child-mortality settings [11,13,20]. Rotavirus vaccines are substantially less immunogenic in high-child-mortality settings when compared with low-child-mortality settings [13,20]. Rotavirus vaccine immunogenicity, represented by serum antirotavirus immunoglobulin A (IgA) antibodies, has been shown to vary by country and is inversely associated with mortality rate in children under 5 years of age at the country level [21].

Reasons for this disparate immunogenicity and efficacy/effectiveness are poorly understood [22,23]. Individual-level host characteristics may play a central role in vaccine immunogenicity by reducing the effective vaccine virus titer (plaque-forming units) delivered to the intestines or reducing the immune response to vaccination [22,24]. For example, immunoglobulin G antibodies received from breast milk might decrease the effective vaccine virus titer delivered to the gut, whereas malnutrition may impair an infant’s immune response to the vaccine [22]. Additional factors that may compromise vaccine performance relate to genetic susceptibility to rotavirus [25–28], environmental enteropathy (chronic inflammation) [29], and differences in force of infection [13,22,30].

One potentially modifiable factor relates to the interaction with live oral poliovirus vaccine (OPV). Poliovirus vaccines are generally administered on the same infant immunization schedule as rotavirus vaccines. In low-child-mortality settings, inactivated poliovirus vaccine (IPV) is primarily administered, whereas in high-child-mortality settings, live OPV is used [31]. OPV administered concomitantly with rotavirus vaccine has been shown to reduce rotavirus seroconversion [32–36]. Early clinical trials suggested that this inhibitory effect may be strongest with the first rotavirus dose, with more modest, statistically nonsignificant differences observed after completion of the full rotavirus vaccine course [37]. However, more recent evidence suggests that interference from OPV—be it monovalent, bivalent, or trivalent—may persist after two doses of rotavirus vaccine [32,35]. If OPV does substantially interfere with RV1, the global strategy to withdraw trivalent OPV, as implemented in 2016, and then OPV entirely [38] has the potential to improve rotavirus vaccine performance.

Developing strategies to improve vaccine performance first requires identification of individual-level factors associated with immune response. Isolating such factors across settings may highlight potentially modifiable vaccine strategies or interventions for enhancing vaccine performance and further reducing the burden of rotavirus disease. Rotavirus vaccine clinical trials were powered to assess vaccine efficacy; however, they were not specifically designed to identify individual-level factors associated with vaccine response. In this study, we pooled data from 22 clinical trials in 33 countries (5 countries/territories with high, 13 with moderately low, and 15 with very low child mortality) to identify a range of individual-level characteristics that contribute to RV1 immunogenicity measured via serum antirotavirus IgA in high- and low-child-mortality settings controlling for individual- and country-level factors. A central hypothesis was that OPV administered concomitantly with rotavirus vaccine doses reduced rotavirus vaccine immunogenicity.

## Methods

### GSK clinical trial data

We pooled individual-level data from infants (5–17 weeks of age) enrolled in GSK’s Phase II and III clinical trials of the RV1 vaccine initiated during 2000–2010 (Table 1). RV1 is an oral two-dose vaccine based on a live, attenuated human rotavirus strain (G1P[8]). GSK recommends that the first dose be administered beginning at 6 weeks of age and the second dose be given after an interval of 4 or more weeks and by 24 weeks of age [39].

**Table 1:** Trial characteristics by GSK trial identification number.

GSK trial number	Study sites	Study phase	Age at dose 1 (weeks)	Vaccinated (*n*)	Serology (*n*)	
					Pre-Vacc.	Post-Vacc.
107625	Japan	3	6–14	492	34	34
444563/023	Argentina, Brazil, Chile, Colombia, Dominican Republic, Finland, Honduras, Mexico, Nicaragua, Panama, Peru, Venezuela	3	6–13	29,753	393	393
444563/024	Argentina, Brazil, Colombia, Dominican Republic, Honduras, Panama	3	6–12	4,234	0	176
444563/028/029/030	Singapore, Hong Kong, Taiwan	3	6–17	5,215	115	115
102247	Czech Republic, Finland, France, Germany, Italy, Spain	3	6–14	2,613	787	787
102248	Malawi, South Africa	3	5–10	2,803	221	2,295
113808	China	3	6–16	1,518	391	391
444563/007	Singapore	2	11–17	1,737	453	454
444563/004	Finland	2	6–12	249	209	209
444563/005	Canada, United States	2	6–12	372	239	270
444563/006	Brazil, Mexico, Venezuela	2	6–12	1,498	427	432
444563/013	South Africa	2	5–10	337	262	264
105722	Vietnam	3	6–10	281	249	249
103792	India	3	8–10	173	115	115
444563/033	Colombia, Mexico, Peru	3	6–12	683	466	468
106481	France, Poland, Portugal, Spain	3	6–14	655	147	147
103478	Republic of Korea	3	6–12	99	48	48
101555	Philippines	2	6–12	95	76	76
109216	Philippines	2	5–10	292	240	240
103992	Bangladesh	2	5–7	193	134	135
Total				53,292	5,006	7,298

Abbreviation: GSK, GlaxoSmithKline; Vacc., vaccine

**Fig 1:**
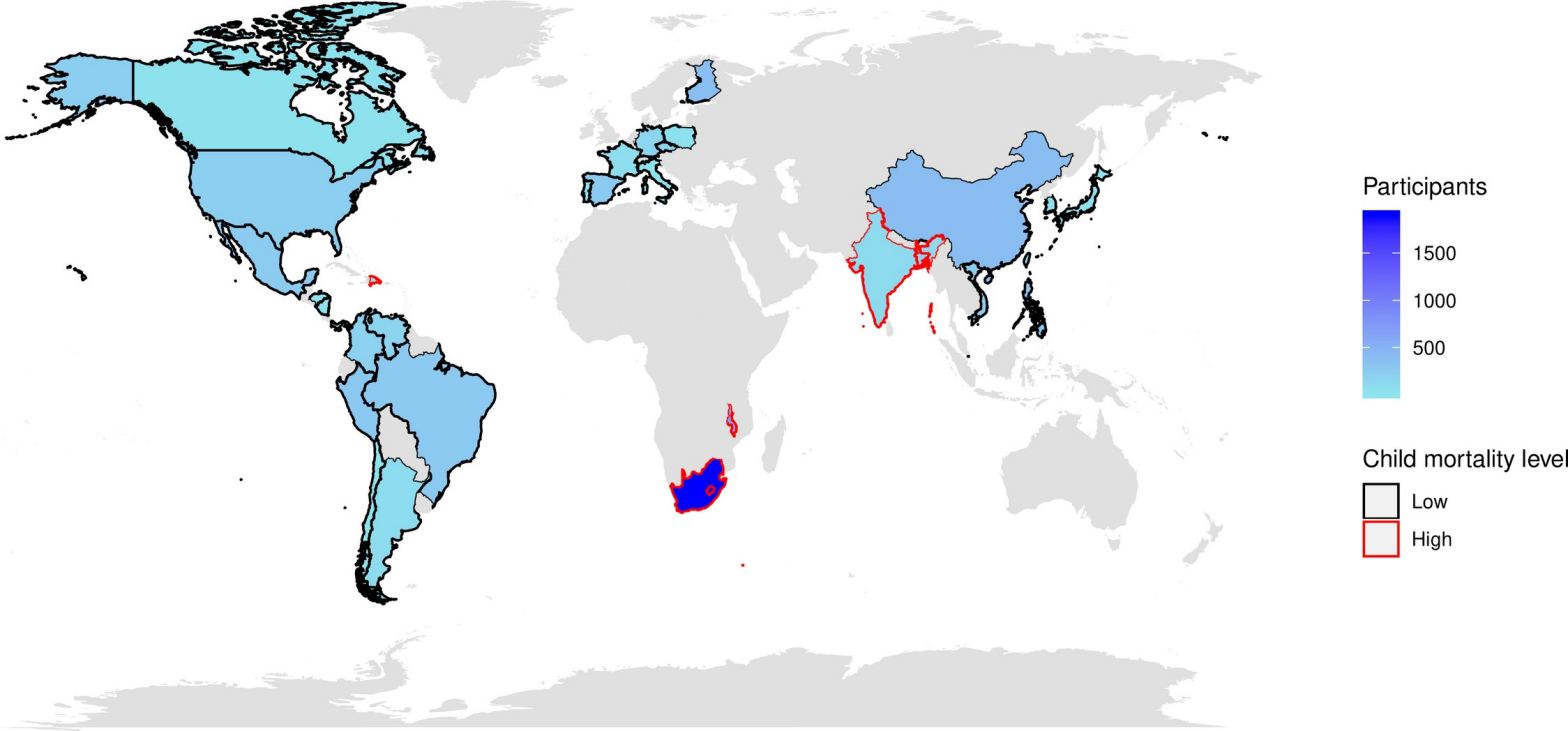
Clinical trial sites included in the analysis. Color shading represents the number of participants from each country. Country outline indicates a country’s child mortality classification (see S1 Table for details). For the primary analysis, countries categorized as “very low” and “moderately low” child mortality were combined and categorized as “low” child mortality. Map created using the “rworldmap” package in R.

The 22 trials included were randomized, double-blind, placebo-controlled trials conducted in a total of 33 countries/territories, including 5 with high, 13 with moderately low, and 15 with very low child mortality (Fig 1). The child mortality strata categories were based on 2004 under-five mortality rates as previously described (S1 Table) [40,41]. Settings with moderately low and very low child mortality were combined and categorized as “low” child mortality for the primary analyses. Clinical research protocols across trials were similar in terms of data collection techniques, vaccine administration, and immunogenicity outcome measures (Table 2). Since the primary aim of the analysis was to examine factors associated with rotavirus vaccine immunogenicity, data were limited to trial participants who received RV1 (*n* = 53,292, placebo groups excluded). Data were further restricted to infants whose trial participation was completed according to protocol (classified by GSK) and who participated in the rotavirus immunogenicity substudies of the trials (*n* = 8,309). Lastly, infants who had serum sample collection approximately 4–12 weeks from receipt of his/her last rotavirus vaccine dose were included (*n* = 7,298).

**Table 2:** Similarities across GSK trial protocols and definitions.

Category	Study protocol and definitions
Inclusion criteria	Male and female infants
	Healthy subjects^a^ free of all obvious health problems (established by medical history and physical exam)
Exclusion and/or elimination criteria	Use of investigational or nonregistered product (drug or vaccine) ≤30 days prior to study vaccine dose
	Planned administration of a vaccine not foreseen by the study protocol ≤14 days of study vaccine dose
	Chronic administration (defined as >14 days) of immunosuppressants anytime since birth
	Any confirmed or suspected immune-suppressive or deficient condition based on medical history and exam
	Significant history of chronic gastrointestinal disease^b^
	History of allergic reaction to any vaccine component
	Acute disease, defined as the presence of a moderate or severe illness with or without fever, at the time of enrollment (warrants deferral of vaccination)^c^
	Administration of immunoglobulins and/or blood product since birth or planned administration during the study
Vaccine	GSK RIX 4414 HRV vaccine
	Vaccinated arm with viral suspension of ≥10^6.0^ CCID_50_^d^
	Doses administered 1–2 months apart
Medical exam and history	Medical exam and history obtained at enrollment
	Concomitant medications/vaccinations, history of medication/vaccination recorded at study visits
	Anthropometric measurements obtained
Serology	Collected 1–2 months after final vaccine dose
	Samples tested via ELISA, assay cutoff of antirotavirus IgA ≥ 20 U/mL

Studies varied in their other exclusion criteria. Some studies excluded individuals based on a history of congenital/hereditary immunodeficiencies or chronic diseases, history of use of experimental rotavirus vaccination, previous routine vaccinations at birth, gastroenteritis in the 7 days preceding the first study vaccination, and/or history of other infectious diseases.

^a^Study 106481 included “medically stable” preterm infants.

^b^Not specified for study 109216.

^c^Not specified for study 444563/023/024/028.

^d^Highest viral suspensions of 10^4.7^ and 10^5.8^ median CCID_50_ in trials 444563/004 and 444563/006, respectively.

Abbreviations: CCID_50_, 50% cell culture infective dose; ELISA, enzyme-linked immunosorbent assay; GSK, GlaxoSmithKline; HRV, human rotavirus; IgA, immunoglobulin A

### Explanatory variables, covariates, and end points of interest

All available data for trial participants were provided by GSK, and explanatory variables/covariates were selected for inclusion in the analysis based on existing literature [22,24]. GSK data were supplemented with country-level data on gross domestic product (GDP) per capita in 2004 USD [42,43] to represent a country’s level of development and 2004 under-five mortality rates [44–46]. These country-level variables were considered in an effort to capture potential confounders that remained unmeasured despite the individual-level factors available.

Two standard markers for immune response [47,48] were analyzed as outcomes. First, in accordance with prespecified trial definitions [49,50], seroconversion was defined as the appearance of serum antirotavirus IgA antibodies (i.e., concentrations ≥20 U/mL) in subjects initially (prior to the first rotavirus dose) seronegative. The second end point of interest was serum antirotavirus IgA titer among infants who seroconverted. In all trials, postvaccine antirotavirus titer IgA data were collected approximately 4–12 weeks after the last administration of rotavirus vaccine and were measured using enzyme-linked immunosorbent assay (ELISA) techniques [51].

### Statistical analysis

Regression models were fit to estimate the effects of individual- and country-level factors on vaccine immunogenicity outcomes while controlling for potential confounders. Mixed-effect logistic regression and mixed-effect linear regression models were used to analyze the antirotavirus IgA seroconversion (dichotomous) and antirotavirus IgA antibody titer (continuous, log transformed) outcomes, respectively. Basic formulas for each model and our detailed analysis plan are shown in S1 Text. Models were fit using the “lme4” package in R software.

The data used for modeling antirotavirus IgA seroconversion included all infants who were either confirmed to be seronegative prior to vaccination via serology sample or who did not have prevaccine serology data available. Prior confirmed rotavirus gastroenteritis was an exclusion criterion in a majority of the included trials, so for the primary analysis in this study, infants without prevaccine serology samples were assumed to be seronegative. The data for modeling antirotavirus IgA titer were restricted to infants who were seropositive after vaccination.

The modeling strategy for both outcomes began with variable specification, incorporating all individual-level characteristics as explanatory variables and controlling for potential country-level covariates selected based on variable distributions and existing literature. Individual-level variables in the initial model included time from last rotavirus dose to serology sample/outcome measure, number of rotavirus vaccine doses, age at first vaccine dose (weeks), vaccine concentration, sex, length-for-age z-score (LAZ) to represent nutritional status, and concomitant OPV. Country-level variables in the initial model included GDP, under-five mortality rate, and child mortality stratum (dichotomous). Because our analysis was restricted to infants who participated in the trials according to protocol, the only instances of missing data were related to LAZ; height was not included in the protocols of a few studies, and infants without these measurements were excluded in the models in which LAZ was used as a predictor (seroconversion models: *n* = 691 [9%]; IgA titer models: *n* = 525 [10%]). Random effects were employed using a random intercept for each trial to account for potentially unmeasured differences between trial protocols or environments. The initial model included all explanatory variables of interest and incorporated interaction terms between each main effect and child mortality stratum. The model was applied to combined data from all child mortality strata, and backward elimination was then conducted using an α = 0.10 cutoff for variable inclusion, maintaining a hierarchically well-formulated model throughout.

Next, models were refined by investigating alternative measures of the included variables based on possible relationships identified in bivariate analyses. For example, higher-order terms for continuous variables (such as age and age-squared) were considered when appropriate, and more-detailed stratification of categorical variables was tested. Backward elimination was subsequently performed after each variable was modified. When initial models were prohibitively large and issues with model convergence were encountered, the most informative end models resulting from previous backward elimination procedures were used as the starting point for investigation of refined measures. After all variables were explored, the most parsimonious model with all relevant variables and covariates was selected as the final model using Akaike information criterion (AIC) criteria (lower AIC indicating a better model) and assessed for multicollinearity using variance inflation factors (VIFs). Population attributable fraction and attributable fraction among the exposed were then calculated for variables of interest.

We examined whether the relationships identified using the full, combined dataset remained consistent within each mortality stratum; this would indicate that use of the combined dataset captured differences beyond those driven by child mortality stratum alone. To this end, a sensitivity analysis was conducted by fitting the final seroconversion model separately to data stratified by high- and low-child-mortality settings, as well as settings with moderately low and very low child mortality. In addition, the final seroconversion model was tested using a subset of the data made up of infants who were confirmed to be seronegative prior to vaccination. This subanalysis was conducted to confirm that results produced from analysis of the full seroconversion dataset were consistent with results using infants with both pre- and postvaccine serology. If consistent, this would suggest that inclusion of infants without prevaccine serology data was not causing substantial bias in our estimates. Lastly, a small number of infants received OPV with only their first or second rotavirus vaccine dose but not both. The final seroconversion model was rerun excluding these infants to simplify the OPV variable into three levels: (1) OPV not received concomitantly with the first and second rotavirus doses, (2) OPV received concomitantly with both rotavirus doses, and (3) no OPV received.

### Ethical approval

All identifiers for trial participants were deidentified and all dates obfuscated by GSK prior to data sharing. This study was determined not to meet the definition of research with human subjects by the Emory University Institutional Review Board. The Centers for Disease Control and Prevention (CDC) was nonengaged.

## Results

Data were available for analysis on 7,298 infants whose postvaccine rotavirus serology data were collected 4–12 weeks after receipt of the final vaccine dose (87.8% of the rotavirus immunogenicity cohort). Of these infants, 39.0% (*n* = 2,849) were from high-child-mortality settings. A total of 18 infants (3 from high-child-mortality settings) were excluded from analysis due to prevaccine serology data indicating these children had prior rotavirus infection (i.e., antirotavirus IgA titer of ≥20 U/mL prior to the first rotavirus vaccine dose). All other infants had either confirmed seronegative status (4,988, 68.5%) or did not have serology data available (2,292, 31.5%) and were assumed to be seronegative based on trial protocol. Data on 7,280 and 5,161 infants were included in the antirotavirus IgA seroconversion and antirotavirus IgA titer modeling, respectively. A basic comparison of the analysis cohort for this study to the entire vaccinated cohort from all 22 trials suggests the populations were demographically similar in terms of sex (analysis cohort versus entire vaccinated cohort: 49.7% versus 49.1% female), height at first dose (56.6 versus 57.4 cm), weight at first dose (5.2 versus 6.5 kilograms), and age at first vaccine dose (9.4 versus 8.8 weeks).

All infants received either two or three doses of rotavirus vaccine (Table 3); nearly all infants in low-child-mortality settings received two doses (99.1%), whereas over half of infants in high-child-mortality settings received a third dose because of study protocols (55.4%). In low-child-mortality settings, one-quarter of infants received a reduced concentration of the vaccine (viral suspension <10^6.0^ 50% cell culture infective dose [CCID_50_]), whereas all infants from high-child-mortality settings received a “standard” concentration (viral suspension ≥10^6.0^ CCID_50_).

**Table 3:** Vaccine and individual- and country-level characteristics of infants from 22 trials conducted in 33 countries/territories beginning in 2000–2010.

	Child mortality setting		
Characteristic	All (*N* = 7,280)	Low (*N* = 4,434)	High (*N* = 2,846)
Vaccine characteristics, *n* (%)			
Standard vaccine concentration^a^	6,208 (85.3)	3,362 (75.8)	2,846 (100.0)
2 rotavirus doses (versus 3 doses)	5,971 (82.0)	4,393 (99.1)	1,578 (55.4)
Individual-level characteristics, *n* (%)			
Female	3,618 (49.7)	2,194 (48.5)	1,424 (50.0)
Length-for-age z-score			
Not stunted	5,604 (77.0)	3,433 (77.4)	2,171 (76.3)
Stunted	588 (8.1)	215 (4.8)	373 (13.1)
Severely stunted	397 (5.5)	135 (3.0)	262 (9.2)
Missing	691 (9.5)	651 (14.7)	40 (1.4)
OPV concomitant with rotavirus dose			
Neither dose 1 nor dose 2	1,835 (25.2)	1,614 (36.4)	221 (7.8)
Dose 1 only	14 (0.2)	3 (0.1)	11 (0.4)
Dose 2 only	26 (0.4)	18 (0.4)	8 (0.3)
Both dose 1 and dose 2	3,384 (46.5)	778 (17.5)	2,606 (92.3)
No OPV received	2,021 (27.8)	2,021 (45.6)	0 (0.0)
Individual-level characteristics, median (IQR)			
Age at first rotavirus dose (weeks)	9 (7, 11)	9 (8, 12)	9 (6, 11)
Time from last rotavirus dose to postvaccine serology (weeks)	5 (5, 8)	8 (5, 9)	5 (5, 5)
Age at postvaccine serology (weeks)	22 (21, 26)	24 (21, 27)	21 (20, 22)
Country-level characteristics, median (IQR)			
GDP (2004, in USD)^b^	4,745 (1,509, 15,356)	7,311 (2,448, 27,405)	4,745 (461, 4,745)
Under-five mortality rate ^c^	27 (8, 85)	19 (5, 26)	85 (85, 85)
Serology outcomes			
Seropositive after vaccination^d^, *n* (%)	5,161 (70.9)	3,411 (76.9)	1,750 (61.5)
Postvaccine antirotavirus IgA titer among seroconverted, geometric mean (SD)	226 (4)	240 (4)	199 (4)

^a^Standard = viral suspension of ≥10^6.0^ CCID_50_, low = viral suspension of <10^6.0^ CCID_50_.

^b^GDP is a country-level parameter in models, assigned and calculated in the table at the individual level (4,745 was the GDP for South Africa, where a large number of study participants were located).

^c^Under-five mortality rate is a country-level parameter presented in the table at the individual level and defined as deaths among children under 5 years of age per 1,000 live births.

^d^Seropositive status defined as antirotavirus IgA titer ≥20 U/mL.

Abbreviations: CCID_50_, 50% cell culture infective dose; GDP, gross domestic product; IgA, immunoglobulin A; IQR, interquartile range; OPV, oral poliovirus vaccine; SD, standard deviation

No association with seroconversion was identified for vaccine concentration, sex, age at first rotavirus vaccine dose, time from last rotavirus vaccine dose to postvaccine serology sample, and age at postvaccine serology sample in unadjusted analyses (Tables 4 and 5). In the unadjusted analysis, infants who received three rotavirus vaccine doses were less likely to seroconvert compared with those who received two doses (unadjusted odds ratio [OR] = 0.57, 95% confidence interval [CI] 0.50–0.64, *p* < 0.001), as nearly three-quarters (73.6%) of children who received three doses were from high-child-mortality settings where seroconversion was less common. Those who received OPV concomitantly with both their first and second doses of rotavirus vaccine had reduced odds of seroconversion compared with those who did not receive OPV concomitantly (unadjusted OR = 0.58, 95% CI 0.51–0.66, *p* < 0.001). A similar relationship was observed for infants who received OPV concomitantly with only their first or second rotavirus dose, though the sample size for these two categories was very small. Child mortality status (OR comparing high-child-mortality settings to low-child-mortality settings = 0.48, 95% CI 0.43–0.53, *p* < 0.001) and under-five mortality rate (OR for each one-unit increase in under-five mortality rate [log scale] = 0.69, 95% CI 0.66–0.72, *p* < 0.001) were negatively associated with seroconversion, whereas GDP was positively associated with seroconversion (OR for each one-unit increase in GDP [log scale] = 1.24, 95% CI 1.20–1.28, *p* < 0.001), as expected. The unadjusted associations between each variable and vaccine titer were generally similar to those observed for seroconversion.

**Table 4:** Categorical vaccine and individual- and country-level characteristics by antirotavirus IgA seroconversion and antirotavirus IgA titer outcomes.

	Seroconversion (*N* = 7,280)			IgA titer (*N* = 5,161)		
Characteristic	*n* seroconverted^a^/*N* (%)	Unadjusted	*p*-Value^b^	Median titer (IQR)	Unadjusted	*p*-Value^c^
		OR (95% CI)			β (95% CI)	
Vaccine characteristics						
Vaccine concentration						
Standard (ref)^d^	4,390/6,208 (71)	1.00 (ref)		211 (74, 621)	1.00 (ref)	
Low	771/1,072 (72)	1.06 (0.92–1.23)	0.422	202 (83, 490)	0.92 (0.83–1.02)	0.093
Number of rotavirus doses						
2 (ref)	4,367/5,971 (73)	1.00 (ref)		213 (78, 608)	1.00 (ref)	
3	794/1,309 (61)	0.57 (0.50–0.64)	<0.001	175 (62, 541)	0.91 (0.82–1.01)	0.063
Individual-level characteristics							
Sex						
Female (ref)	2,549/3,618 (70)	1.00 (ref)		215 (79, 622)	1.00 (ref)	
Male	2,612/3,662 (71)	0.96 (0.87–1.06)	0.412	201 (73, 577)	0.94 (0.87–1.01)	0.096
Length-for-age z-score						
Not stunted (ref)	3,979/5,604 (71)	1.00 (ref)		210 (76, 598)	1.00 (ref)	
Stunted	393/588 (67)	0.84 (0.70–1.01)	0.050	177 (67, 624)	0.95 (0.83–1.10)	0.503
Severely stunted	264/397 (66)	0.83 (0.67–1.03)	0.083	202 (69, 518)	0.95 (0.80–1.12)	0.541
Missing	525/691 (76)	--		211 (74, 565)	--	
OPV concomitant with rotavirus dose^e^						
Neither dose 1 nor dose 2 (ref)	1,358/1,835 (74)	1.00 (ref)		182 (74, 501)	1.00 (ref)	
Dose 1 only	7/14 (50)	0.35 (0.12–1.01)	0.051	324 (104, 531)	1.03 (0.19–1.26)	0.139
Dose 2 only	17/26 (65)	0.66 (0.29–1.50)	0.324	114 (62, 511)	0.82 (0.34–1.35))	0.272
Both dose 1 and dose 2	2,109/3,384 (62)	0.58 (0.51–0.66)	<0.001	177 (62, 577)	1.04 (0.95–1.14)	0.411
No OPV received	1,670/2,021 (83)	1.67 (1.43–1.95)	<0.001	260 (101, 687)	1.32 (1.46–1.82)	<0.001
Country-level characteristics						
Child mortality status						
Low child mortality (ref)	3,411/4,434 (77)	1.00 (ref)		115 (6)^f^	1.00 (ref)	<0.001
High child mortality	1,750/2,846 (61)	0.48 (0.43–0.53)	<0.001	63 (6)^f^	0.83 (0.77–0.90)	

^a^Defined as serum antirotavirus IgA titer ≥20 U/mL.

^b^Determined by chi-squared test.

^c^Determined by *t* test.

^d^Standard = viral suspension of ≥10^6.0^ CCID_50_, low = viral suspension of <10^6.0^ CCID_50_.

^e^Of the 1,309 infants who received three doses of rotavirus vaccine, nearly all (97%) received it concomitantly with OPV.

^f^Geometric mean.

Abbreviations: CCID_50_, 50% cell culture infective dose CI, confidence interval; IgA, immunoglobulin A; IQR, interquartile range; OPV, oral poliovirus vaccine; OR, odds ratio; ref, reference group

**Table 5:** Continuous individual- and country-level characteristics by antirotavirus IgA seroconversion and antirotavirus IgA titer outcomes.

	Seroconversion (*N* = 7,280)				IgA titer (*N* = 5,161)		
Characteristic	Seroconverted, median (IQR)	Did not seroconvert, median (IQR)	Unadjusted OR (95% CI)	*p*-Value^a^	Median (IQR)	Unadjusted mean difference, β (95% CI)	*p*-Value^b^
Individual-level characteristics							
Age at first rotavirus dose (weeks)	9 (7, 12)	9 (7, 11)	1.07 (1.05–1.09)	<0.001	9 (7, 12)	1.00 (0.99–1.01)	0.838
Time from last rotavirus dose to postvaccine serology (weeks)	5 (5, 9)	5 (5, 8)	1.04 (1.01–1.06)	0.005	5 (5, 9)	0.97 (0.95–0.99)	<0.001
Age at postvaccine serology (weeks)	23 (21, 26)	22 (20, 25)	1.06 (1.05–1.08)	<0.001	23 (21, 26)	1.00 (0.99–1.01)	0.925
Country-level characteristics							
GDP^c^	4,745 (2,448, 24,919)	4,745 (1,079, 4,745)	1.24 (1.20–1.28)*	<0.001	4,745 (2,448, 24,919)	1.05 (1.02–1.08)*	<0.001
Under-five mortality rate^c^	26 (5, 85)	70 (22, 85)	0.69 (0.66–0.72)*	<0.001	26 (5, 85)	0.81(0.76–0.87)*	<0.001

^a^Determined by chi-squared test.

^b^Determined by *t* test.

^c^Country-level parameter in models, assigned and calculated in the table at the individual level (4,745 was the GDP for South Africa, where a large number of study participants were located).

*Predictor is on log scale.

Abbreviations: CI, confidence interval; GDP, gross domestic product; IgA, immunoglobulin A; IQR, interquartile range; OR, odds ratio

Individual-level characteristics frequently differed by setting (Table 3). Over 20% of infants in high-child-mortality settings were stunted or severely stunted, whereas fewer than 10% were in low-child-mortality settings (*p* < 0.001). All infants in high-child-mortality settings received OPV, and over 90% of infants who received OPV received it concomitantly with both their first and second doses of rotavirus vaccine. In contrast, nearly half of infants in low-child-mortality settings did not receive any OPV (because IPV was more commonly administered in these settings). The percentage of children who seroconverted among the OPV categories ranged from 50% among those who received OPV concomitantly with only their first rotavirus vaccine dose to 83% among infants who did not receive OPV (Table 4). The median age at receipt of the first rotavirus dose was 9 weeks (interquartile range [IQR] = 7, 11) and infants were a median of 22 weeks of age (IQR = 21, 26) when their postvaccine serology sample was collected. The time from receipt of the last rotavirus dose to serology sample was slightly longer for infants in low-child-mortality settings (Table 3, median of 8 weeks, IQR = 5, 9) compared with infants in high-child-mortality settings (5 weeks, IQR = 5, 5, *p* < 0.001).

**Fig 2:**
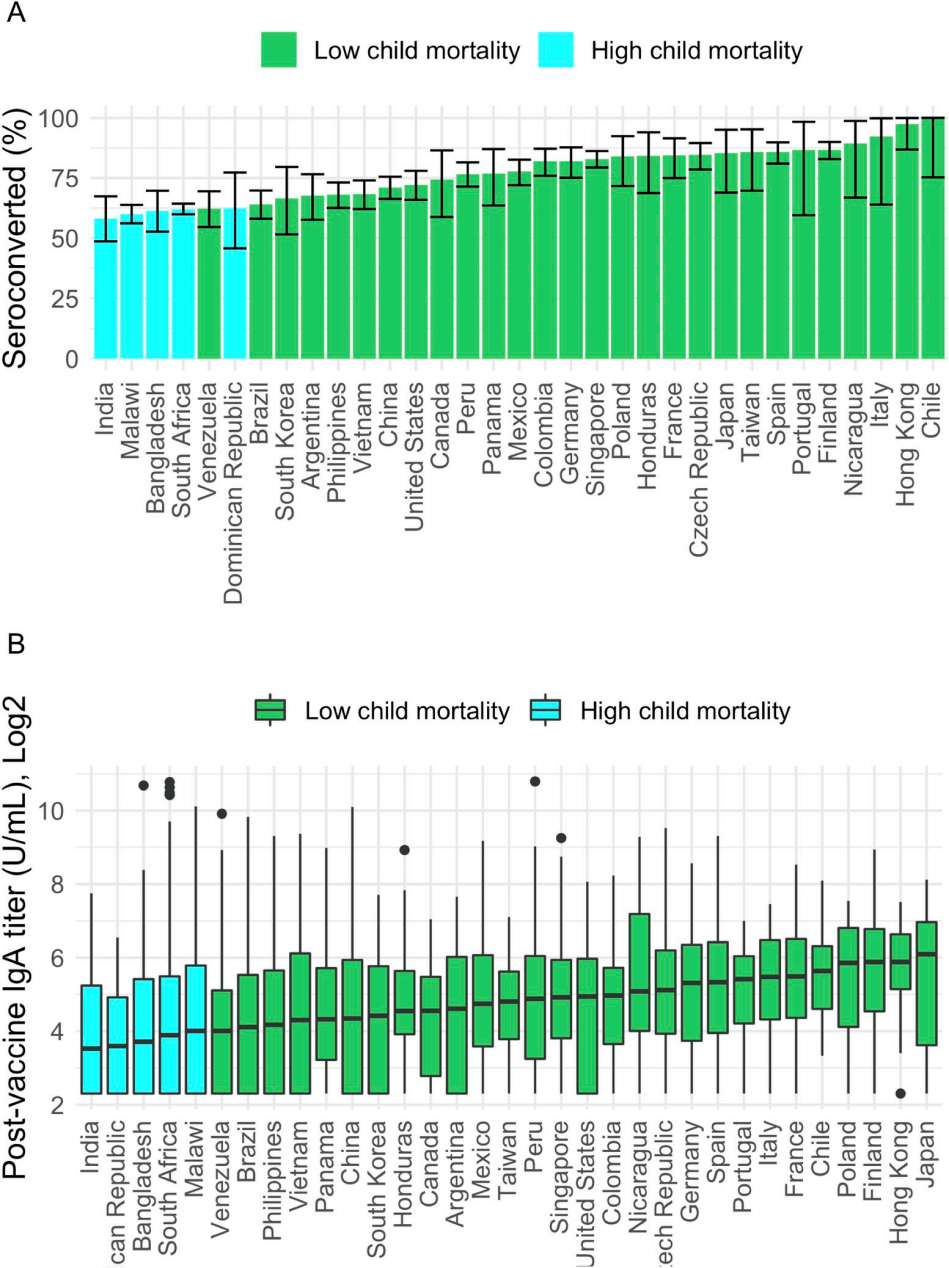
Percent of infants who seroconverted by country and postvaccine antirotavirus IgA titer (U/mL) by country. (A) Colored bars represent the percentage of infants in a given country that seroconverted, defined as a serum antirotavirus IgA titer ≥20 U/mL. Error bars indicate the 95% confidence interval. (B) Colored bars represent median and IQR for postvaccine antirotavirus IgA titer. Lines represent 25th percentile − 1.5*IQR and 75th percentile + 1.5*IQR, with dots representing outliers below or above these lines (Tukey box plot method). IgA, immunoglobulin A; IQR, interquartile range.

A majority of infants (70.9%) were seropositive after vaccination (Table 3), with a higher proportion of infants in low-child-mortality settings seroconverting (76.9%) compared with infants in high-child-mortality settings (61.5%, *p* < 0.001). Antirotavirus IgA seroconversion ranged from 58% in India to over 90% in Hong Kong, Italy, and Chile (Fig 2A). A similar pattern was found with postvaccine antirotavirus IgA titer, in which infants in low-child-mortality settings had geometric mean titers (240, SD = 4) higher than that of infants in high-child-mortality settings (199, SD = 4, *p* < 0.001). Antirotavirus IgA titer ranged from a median of 34 U/mL in India to 443 U/mL in Japan (Fig 2B).

### Antirotavirus IgA seroconversion modeling results

Results of backward elimination and model refinement for antirotavirus IgA seroconversion indicated several main effects and interaction terms contribute to the odds of seroconversion (Table 6). Being in a country with higher GDP and older age at first rotavirus dose were both associated with increased likelihood of antirotavirus IgA seroconversion. In contrast, increased time from last rotavirus dose to serology, low vaccine concentration, and concomitant receipt of OPV with the first and second rotavirus doses were each negatively associated with antirotavirus IgA seroconversion. Infants who received OPV concomitantly with both their first and second doses of rotavirus vaccine had 0.63 times the odds of seroconverting (OR = 0.63, 95% CI 0.47–0.84, *p* = 0.002) compared with infants who received OPV but not concomitantly with either dose. The final model included interaction terms with child mortality strata for both age at first vaccine dose and LAZ score. Sex, the number of rotavirus vaccine doses received, and under-five mortality rate were dropped from the model during backward elimination.

**Table 6:** Final serum antirotavirus IgA seroconversion model developed using both child mortality strata combined (*n* = 6,589).

Individual- or country-level factor	OR (95% CI)	*p*-Value^a^
Time from last rotavirus dose to serology (per week)	0.90 (0.86–0.94)	<0.001
Vaccine concentration ≥10^6.0^	1.00 (ref)	
Vaccine concentration <10^6.0^	0.65 (0.49–0.87)	0.003
OPV concomitant with neither rotavirus dose 1 nor 2	1.00 (ref)	
OPV concomitant with rotavirus dose 1 and 2	0.63 (0.47–0.84)	0.002
OPV concomitant with rotavirus dose 1 only	0.36 (0.12–1.05)	0.062
OPV concomitant with rotavirus dose 2 only	0.66 (0.27–1.57)	0.343
No OPV received	1.13 (0.76–1.69)	0.544
Log(GDP)	1.11 (1.04–1.19)	0.001
Age at first rotavirus dose (weeks)	1.13 (1.08–1.17)	<0.001
Age at first rotavirus dose (weeks)*child mortality setting	0.90 (0.86–0.95)	<0.001
LAZ: stunted or severely stunted	1.00 (ref)	
LAZ: not stunted/severely stunted	1.24 (0.93–1.65)	0.137
LAZ: stunted or severely stunted*child mortality setting	0.67 (0.47–0.95)	0.025
Child mortality setting: low	1.00 (ref)	
Child mortality setting: high	1.62 (0.83–3.14)	0.155

^**a**^Determined by Wald test.

Abbreviations: CI, confidence interval; GDP, gross domestic product; IgA, immunoglobulin A; LAZ, length-for-age z-score; OPV, oral poliovirus vaccine; OR, odds ratio; ref, reference group

Among those who received OPV concomitantly with both the first and second rotavirus vaccine doses, 58.2% (95% CI 18.7%–100.0%) of vaccine nonresponse (i.e., those who did not seroconvert) was due to concomitant OPV administration (attributable fraction among the exposed). At the population level, the attributable fraction depends on the proportion of the population exposed; for example, if 95% of an infant population was administered OPV concomitantly with the first two doses of rotavirus vaccine (an estimate based on WHO’s recommended administration of both vaccines at 6 and 10 weeks of age), over half (53.7%, 95% CI 17.6%–99.7%) of vaccine nonresponses would be attributable to concomitant OPV administration with the first two rotavirus vaccine doses.

When the final antirotavirus IgA seroconversion model was applied to each mortality stratum separately (high, low, moderately low, and very low child mortality), similar results were observed (S2 Table). The negative relationship between OPV concomitant with both rotavirus doses 1 and 2 remained within each mortality setting, though with less precision of the parameter estimate. Similarly, when including only infants confirmed to be seronegative prior to rotavirus vaccination, results were consistent with the original analysis (S3 Table). Infants who received OPV concomitantly with rotavirus dose 1 and dose 2 were less likely to seroconvert than others (OR = 0.62, 95% CI 0.46–0.81, *p* = 0.001). The relationships between other variables and antirotavirus IgA seroconversion displayed only modest changes. Lastly, when the final model was fitted, excluding infants who received OPV concomitantly with only their first or second rotavirus vaccine doses, similar results were observed (S4 Table).

### Antirotavirus IgA titer modeling results

Few factors were found to be significantly associated with antirotavirus IgA titers among those children who seroconverted (Table 7). Even when models were refined to explore alternative measures of various factors and when OPV was forced to remain in the model (based on its importance in seroconversion models), the results varied minimally. Increased time from last rotavirus dose to serology sample was negatively associated with antirotavirus IgA titer. Among infants who seroconverted, concomitant OPV with rotavirus doses 1 and 2 was positively associated with antirotavirus IgA titer (β = 1.28, 95% CI 1.07–1.53, *p* = 0.009), an opposite relationship to that observed among all infants in the seroconversion analysis.

**Table 7:** Final serum antirotavirus IgA titer model developed using both child mortality strata combined (*n* = 5,161).

Individual- or country-level factor	Mean difference	*p*-Value^a^
	β (95% CI)	
Time from last rotavirus dose to serology (per week)	0.92 (0.90–0.95)	<0.001
Sex (ref = female)	0.93 (0.87–1.00)	0.055
OPV concomitant with neither rotavirus dose 1 nor dose 2	1.00 (ref)	
OPV concomitant with rotavirus doses 1 and 2	1.28 (1.07–1.53)	0.009
OPV concomitant with rotavirus dose 1 only	1.32 (0.49–3.58)	0.583
OPV concomitant with rotavirus dose 2 only	0.92 (0.48–1.79)	0.815
No OPV received	0.98 (0.75–1.28)	0.874
Log(GDP)	1.16 (1.06–1.28)	0.001
Log(GDP)*child mortality setting	0.77 (0.70–0.86)	<0.001
Child mortality setting: low	1.00 (ref)	
Child mortality setting: high	0.76 (0.61–0.93)	0.014

^**a**^Determined by *t* test.

Abbreviations: CI, confidence interval; GDP, gross domestic product; IgA, immunoglobulin A; OPV, oral poliovirus vaccine; ref, reference group

## Discussion

We had the unique ability to pool individual-level data from 22 clinical trials and 33 countries/territories to create a dataset that enabled multilevel assessment of RV1 immunogenicity across settings. The resulting dataset was substantially larger than that of any individual trial or other related study [22]. Our findings suggest that OPV given concomitantly with RV1 reduces antirotavirus IgA seroconversion even after two rotavirus vaccine doses. We did not find the same modifiable characteristics to be associated with postvaccine antirotavirus IgA titers among infants who seroconverted, suggesting that such factors may predict whether an infant responds to RV1 but not the intensity of the response given seroconversion.

These data provide robust evidence that infants who received OPV with both the first and second doses of RV1 were substantially less likely to seroconvert when compared with those not receiving OPV concomitantly. This analysis bolsters existing evidence [32–36] that OPV may interfere with rotavirus seroconversion and suggests that OPV may interfere with seroconversion when given with both the first and second rotavirus vaccine doses. Early evidence indicated that the OPV–rotavirus interaction may be strongest for the first rotavirus dose, with more recent evaluations suggesting lower seroconversion rates after completion of the full rotavirus vaccine course [32,33,35,37]. With this much larger and global dataset, our findings provide support for more recent data [32,35] that suggest a second RV1 dose may not compensate for the reduced initial response. Applying our final seroconversion model to each mortality stratum individually and conducting a separate sensitivity analysis restricting data to only infants with confirmed prevaccine seronegative status provided support, as the direction of the relationship remained within each stratum and despite dropping a substantial portion of the study data. This finding is consistent with the hypothesis the OPV interferes with rotavirus vaccine response, and this effect is not explained by socioeconomic differences among the trial sites. Although our study does not allow for assessment of possible mechanisms behind this relationship, it is consistent with hypotheses related to reduced efficiency of viral entry into mucosal cells, poliovirus causing down-regulation of components of the immune system response to rotavirus [32,52], or stimulation of innate immunity resulting in reduced rotavirus-specific immune response.

Overall, these results raise important programmatic considerations for rotavirus vaccination and evolving OPV eradication strategies. Although it may not be pragmatic to intentionally stagger OPV and rotavirus immunization schedules [32], the eventual shift from OPV to IPV may result in sizeable increases in RV1 performance in high-child-mortality settings. This notion is further supported by a prior study from Bangladesh that found that each formulation of OPV (monovalent, bivalent, and trivalent) reduced rotavirus seroconversion when administered concomitantly with rotavirus vaccine. Data on the specific formulation of OPV (trivalent versus bivalent) administered to infants in the trials in our study were not available to us, though it is likely that most, if not all, individuals who received OPV received the trivalent form based on the date of administration and national immunization schedules in these settings at the time of the trials.

GDP is known to be strongly associated with rotavirus seroconversion [21]; however, the effect of GDP is clearly not causal. Rather, GDP serves as a proxy for individual, family, and community factors that are more directly influential in determining vaccine immunogenicity, a portion of which we aimed to identify in this study. We found that GDP remained associated with the probability of seroconversion even after adjusting for child mortality and concomitant OPV vaccination. Comparing the adjusted OR for GDP and seroconversion developed via model selection (Table 6, OR = 1.11, 95% CI 1.04–1.19, *p* = 0.001) to their crude association (Table 5; OR = 1.24, 95% CI 1.20–1.28, *p* < 0.001) suggests that a substantial, though incomplete, portion of the crude association was accounted for with our individual-level factors.

We were unable to identify many modifiable factors associated with postvaccine antirotavirus IgA titers among infants who seroconverted, and the relationships between IgA titer and concomitant OPV were opposite to those expected; this suggests that intensity of a seropositive individual’s immune response to RV1 may be complex. Our unadjusted models demonstrated a negative relationship between OPV concomitantly administered with the first two doses of RV1 and antirotavirus IgA titer. However, given seroconversion and after adjusting for child mortality status, this relationship was no longer observed. These results are similar to those of Ramani and colleagues, who found that OPV coadministered with rotavirus vaccine reduced rotavirus seroconversion but did not reduce antirotavirus IgA titer among those who seroconverted [35]. We observed a wide range in antirotavirus IgA titer, which may be due to a complex combination of individual-level factors not included in this analysis. As such, additional studies are needed to determine whether antirotavirus IgA titers among those who seroconverted may serve as a valuable correlate of protection for identifying the mechanisms resulting in differential vaccine immunogenicity and whether they are directly associated with vaccine effectiveness (i.e., the clinical relevance of IgA titers once seroconversion has been reached).

There are important challenges and limitations with this approach. First, although trial protocols were remarkably similar, trials themselves still differed by location, year, and population. We attempted to account for this variability by including a random effect for trial in our models. The required adherence to study protocol and stringent monitoring necessary for a clinical trial means that the results produced from these analyses may not perfectly reflect the findings that would have occurred under more routine, real-world conditions and should, therefore, be interpreted accordingly. Relatedly, the infants included in the trials were all healthy children, potentially limiting the generalizability of the results. Second, this is a secondary analysis of data previously collected for other primary purposes. We lacked data to control for genetic, maternal, socioeconomic, and environmental factors that likely influence individual-level immune response to vaccination. To mitigate residual confounding from factors such as socioeconomic status or environment, a proxy measure (GDP) was included in the models. Third, of the 7,280 infants included in the analysis, 2,292 (31.5%) did not have prevaccine serology data. We addressed this limitation by conducting a sensitivity analysis in which the antirotavirus seroconversion model was applied to only infants confirmed to be seronegative prior to vaccination, and we found the effect of OPV to be similar. Lastly, our analysis was limited to infants who received RV1, and it is possible that the findings and implications of this research may not be generalizable to the other three rotavirus vaccines prequalified by WHO or others currently in development.

Improving rotavirus vaccine performance requires identification of the factors that contribute to vaccine immunogenicity on the individual level. Although we explored a number of potential factors, our findings highlight the importance of concomitant OPV administration and provide encouraging evidence to suggest OPV withdrawal could improve RV1 performance. The ongoing effort by the Global Polio Eradication Initiative to end OPV use creates an ideal natural experiment to confirm our results in the real world. Vaccine immunogenicity data from infants in settings where OPV is currently in use could be compared with immunogenicity among infants after OPV withdrawal. More important still are evaluations of rotavirus vaccine effectiveness administered before and after OPV withdrawal against the clinical end point of rotavirus gastroenteritis. This research provides important programmatic considerations for improving rotavirus vaccine immunogenicity, particularly reduction in concomitant rotavirus vaccine and OPV administration.

## Supporting information

S1 STROBE Checklist STROBE, Strengthening the Reporting of Observational studies in Epidemiology. (DOCX)

S1 Text Model equations. Basic model formulas for mixed-effect logistic regression and mixed-effect linear regression of log-transformed data for antirotavirus IgA seroconversion (dichotomous) and antirotavirus IgA titer (continuous) outcomes, respectively. IgA, immunoglobulin A.
(DOCX)

S2 Text Analysis plan. (DOCX)

S1 Table Trial sites by child mortality stratum. (DOCX)

S2 Table Final antirotavirus IgA seroconversion model applied to each child mortality stratum individually. IgA, immunoglobulin A.
(DOCX)

S3 Table Final antirotavirus IgA seroconversion model applied to a subset of infants with confirmed seronegative status via antirotavirus IgA serology sample prior to vaccination (*n* = 4,473). IgA, immunoglobulin A.
(DOCX)

S4 Table Final serum antirotavirus IgA seroconversion model excluding infants who received OPV concomitantly with only their first or second rotavirus vaccine doses (*n* = 6,549). IgA, immunoglobulin A; OPV, oral poliovirus vaccine.
(DOCX)

## Abbreviations

AIC: Akaike information criterion
CCID_50_: 50% cell culture infective dose
CDC: Centers for Disease Control and Prevention
CI: confidence interval
ELISA: enzyme-linked immunosorbent assay
GDP: gross domestic product
GSK: GlaxoSmithKline
IgA: immunoglobulin A
IPV: inactivated poliovirus vaccine
IQR: interquartile range
LAZ: length-for-age z-score
OPV: oral poliovirus vaccine
OR: odds ratio
ref: reference group
RV1: monovalent rotavirus vaccine
SD: standard deviation
STROBE: Strengthening the Reporting of Observational studies in Epidemiology
VIF: variance inflation factor
WHO: World Health Organization
