# Supplementary material for: Antirotavirus IgA seroconversion rates in children who receive concomitant oral poliovirus vaccine: A secondary, pooled analysis of Phase II and III trial data from 33 countries
Source: PLoS Med. 2019 Dec 30;16(12):e1003005. doi: 10.1371/journal.pmed.1003005 (PMC6936798; doi:10.1371/journal.pmed.1003005)
Supplement: S1 STROBE Checklist — (DOCX) [file pmed.1003005.s001.docx]

STROBE Statement—Checklist of items that should be included in reports of ***cross-sectional studies***

|  | Item No | Recommendation | Section & Paragraph |
| --- | --- | --- | --- |
| **Title and abstract** | 1 | *(*a) Indicate the study’s design with a commonly used term in the title or the abstract  **Text:**  “a secondary, pooled analysis” | Title |
|  |  | (*b*) Provide in the abstract an informative and balanced summary of what was done and what was found  **Text:**  “Pooled, individual-level data from GlaxoSmithKline’s phase II and III clinical trials of the Rotarix (RV1) vaccine were analyzed, including 7,280 vaccinated infants (5-17 weeks of age at first vaccine dose) from 22 trials and 33 countries/territories (5 high, 13 moderately low, and 15 very low child mortality countries/territories). Two standard markers for immune response were examined including anti-rotavirus IgA seroconversion (defined as the appearance of serum anti-rotavirus IgA antibodies in subjects initially seronegative) and serum anti-rotavirus IgA titer, both collected approximately 4-12 weeks after administration of the last rotavirus vaccine dose. Mixed effect logistic regression and mixed effect linear regression of log-transformed data were used to identify individual and country-level predictors of seroconversion (dichotomous) and antibody titer (continuous), respectively.”  “Infants in high child mortality settings had lower odds of seroconverting compared to infants in low child mortality settings (OR = 0.48, 95% CI = 0.43, 0.53, p < 0.001). Similarly, among those who seroconverted, infants in high child mortality settings had lower IgA titers compared to infants in low child mortality settings (β = 0.83, 95% CI = 0.77, 0.90, p < 0.001). Infants who received OPV concomitantly with both their first and second doses of rotavirus vaccine had 0.63 times the odds of seroconverting (OR = 0.63, 95% CI = 0.47, 0.84, p = 0.002) compared to infants who received OPV but not concomitantly with either dose. In contrast, among infants who seroconverted, OPV concomitantly administered with both the first and second rotavirus vaccine doses was found to be positively associated with anti-rotavirus IgA titer (β = 1.28, 95% CI = 1.07, 1.53, p = 0.009).” | Abstract, Methods and Findings section  Abstract, Methods and Findings section |
| Introduction | | | |
| Background/rationale | 2 | Explain the scientific background and rationale for the investigation being reported  **Text:**  “Developing strategies to improve vaccine performance first requires identification of individual-level factors associated with immune response. Isolating such factors across settings may highlight potentially modifiable vaccine strategies or interventions for enhancing vaccine performance and further reducing the burden of rotavirus disease. Rotavirus vaccine clinical trials were powered to assess vaccine efficacy, however, they were not specifically designed to identify individual-level factors associated with vaccine response.” | Introduction, last paragraph |
| Objectives | 3 | State specific objectives, including any prespecified hypotheses  **Text:**  “In this study, we pooled data from 22 clinical trials in 33 countries (5 high, 13 moderately low, and 15 very low child mortality countries/territories) to identify a range of individual-level characteristics that contribute to RV1 vaccine immunogenicity measured via serum anti-rotavirus IgA in high and low child mortality settings controlling for individual and country-level factors.” | Introduction, last paragraph |
| Methods | | | |
| Study design | 4 | Present key elements of study design early in the paper  **Text:**  “We pooled individual-level data from infants (5-17 weeks of age) enrolled in GSK’s phase II and III clinical trials of the RV1 vaccine initiated during 2000–2010 (Table 1).”  “The 22 trials included were randomized, double-blind, placebo-controlled trials conducted in a total of 33 countries/territories including 5 high, 13 moderately low, and 15 very low child mortality countries (Fig 1). The child mortality strata categories were based on 2004 under 5 mortality rates as previously described (Table S1) [40,41]. Moderately low and very low child mortality settings were combined and categorized as “low” child mortality for the primary analyses.” | Methods, 1^st^ paragraph  Methods, 2^nd^ paragraph |
| Setting | 5 | Describe the setting, locations, and relevant dates, including periods of recruitment, exposure, follow-up, and data collection  **Text:**  “We pooled individual-level data from infants (5-17 weeks of age) enrolled in GSK’s phase II and III clinical trials of the RV1 vaccine initiated during 2000–2010 (Table 1).”  “The 22 trials included were randomized, double-blind, placebo-controlled trials conducted in a total of 33 countries/territories including 5 high, 13 moderately low, and 15 very low child mortality countries (Fig 1).”  “Lastly, infants who had serum sample collection approximately 4-12 weeks from receipt of his/her last rotavirus vaccine dose were included (n = 7,298).” | Methods, 1^st^ paragraph  Methods, 2^nd^ paragraph  Methods, 2^nd^ paragraph |
| Participants | 6 | *(*a) Give the eligibility criteria, and the sources and methods of selection of participants  **Text:**  “Since the primary aim of the analysis was to examine factors associated with rotavirus vaccine immunogenicity, data were limited to trial participants who received RV1 (n = 53,292, placebo groups excluded). Data were further restricted to infants whose trial participation was completed according to protocol (classified by GSK) and who participated in the rotavirus immunogenicity sub-studies of the trials (n = 8,309). Lastly, infants who had serum sample collection approximately 4-12 weeks from receipt of his/her last rotavirus vaccine dose were included (n = 7,298).” | Methods, 2^nd^ paragraph |
| Variables | 7 | Clearly define all outcomes, exposures, predictors, potential confounders, and effect modifiers. Give diagnostic criteria, if applicable  **Text:**  **“**The child mortality strata categories were based on 2004 under 5 mortality rates as previously described (Table S1) [40,41]. Moderately low and very low child mortality settings were combined and categorized as “low” child mortality for the primary analyses.”  “All available data for trial participants were provided by GSK and explanatory variables/covariates were selected for inclusion in the analysis based on existing literature [22,24]. GSK data were supplemented with country-level data on gross domestic product (GDP) per capita in 2004 USD [42,43] to represent a country’s level of development and 2004 under 5 mortality rates [44–46]. These country-level variables were considered in an effort to capture potential confounders that remained unmeasured despite the individual-level factors available.”  “Two standard markers for immune response [47,48] were analyzed as outcomes. First, in accordance with pre-specified trial definitions [49,50], seroconversion was defined as the appearance of serum anti-rotavirus IgA antibodies (i.e. concentrations ≥20 U/mL) in subjects initially (prior to the first rotavirus dose) seronegative. The second endpoint of interest was serum anti-rotavirus IgA titer among infants who seroconverted. In all trials, post-vaccine anti-rotavirus titer IgA data were collected approximately 4-12 weeks after the last administration of rotavirus vaccine and were measured using enzyme-linked immunosorbent assay (ELISA) techniques [51].” | Methods, 2^nd^ paragraph  Methods, 3^rd^ paragraph  Methods, 4^th^ paragraph |
| Data sources/ measurement | 8* | For each variable of interest, give sources of data and details of methods of assessment (measurement). Describe comparability of assessment methods if there is more than one group  **Text:**  “The child mortality strata categories were based on 2004 under 5 mortality rates as previously described (Table S1) [40,41]. Moderately low and very low child mortality settings were combined and categorized as “low” child mortality for the primary analyses.”  “All available data for trial participants were provided by GSK and explanatory variables/covariates were selected for inclusion in the analysis based on existing literature [22,24]. GSK data were supplemented with country-level data on gross domestic product (GDP) per capita in 2004 USD [42,43] to represent a country’s level of development and 2004 under 5 mortality rates [44–46]. These country-level variables were considered in an effort to capture potential confounders that remained unmeasured despite the individual-level factors available.”  “Two standard markers for immune response [47,48] were analyzed as outcomes. First, in accordance with pre-specified trial definitions [49,50], seroconversion was defined as the appearance of serum anti-rotavirus IgA antibodies (i.e. concentrations ≥20 U/mL) in subjects initially (prior to the first rotavirus dose) seronegative. The second endpoint of interest was serum anti-rotavirus IgA titer among infants who seroconverted. In all trials, post-vaccine anti-rotavirus titer IgA data were collected approximately 4-12 weeks after the last administration of rotavirus vaccine and were measured using enzyme-linked immunosorbent assay (ELISA) techniques [51].” | Methods, 2^nd^ paragraph  Methods, 3^rd^ paragraph  Methods, 4^th^ paragraph |
| Bias | 9 | Describe any efforts to address potential sources of bias  **Text:**  “GSK data were supplemented with country-level data on gross domestic product (GDP) per capita in 2004 USD [42,43] to represent a country’s level of development and 2004 under 5 mortality rates [44–46]. These country-level variables were considered in an effort to capture potential confounders that remained unmeasured despite the individual-level factors available.”  “We examined whether the relationships identified using the full, combined dataset remained consistent within each mortality stratum; this would indicate that use of the combined dataset captured differences beyond those driven by child mortality stratum alone. To this end, a sensitivity analysis was conducted by fitting the final seroconversion model separately to data stratified by high and low child mortality settings, as well as moderately low and very low child mortality settings.”  “In addition, the final seroconversion model was tested using a subset of the data comprised of infants who were confirmed to be seronegative prior to vaccination. This sub-analysis was conducted to confirm that results produced from analysis of the full seroconversion dataset were consistent with results using infants with both pre- and post-vaccine serology. If consistent, this would suggest that inclusion of infants without pre-vaccine serology data was not causing substantial bias in our estimates.” | Methods, 3^rd^ paragraph  Methods, 2^nd^ to last paragraph  Methods, 2^nd^ to last paragraph |
| Study size | 10 | Explain how the study size was arrived at  **Text:**  “We pooled individual-level data from infants (5-17 weeks of age) enrolled in GSK’s phase II and III clinical trials of the RV1 vaccine initiated during 2000–2010 (Table 1).”  “Since the primary aim of the analysis was to examine factors associated with rotavirus vaccine immunogenicity, data were limited to trial participants who received RV1 (n = 53,292, placebo groups excluded). Data were further restricted to infants whose trial participation was completed according to protocol (classified by GSK) and who participated in the rotavirus immunogenicity sub-studies of the trials (n = 8,309). Lastly, infants who had serum sample collection approximately 4-12 weeks from receipt of his/her last rotavirus vaccine dose were included (n = 7,298).”  “A total of 18 infants (3 from high child mortality settings) were excluded from analysis due to pre-vaccine serology data indicating these children had prior rotavirus infection (i.e. anti-rotavirus IgA titer of ≥20 U/mL prior to the first rotavirus vaccine dose)… Data on 7,280 and 5,161 infants were included in the anti-rotavirus IgA seroconversion and anti-rotavirus IgA titer modeling, respectively.” | Methods, 1^st^ paragraph  Methods, 2^nd^ paragraph  Results, 1^st^ paragraph |
| Quantitative variables | 11 | Explain how quantitative variables were handled in the analyses. If applicable, describe which groupings were chosen and why  **Text:**  “Individual-level variables in the initial model included: time from last rotavirus dose to serology sample, number of rotavirus vaccine doses, age at first vaccine dose (weeks), vaccine concentration, sex, length-for-age z-score (LAZ) to represent nutritional status, and concomitant OPV. Country-level variables in the initial model included: GDP, under 5 mortality rate, and child mortality stratum (dichotomous).” | Methods, 7^th^ paragraph |
| Statistical methods | 12 | *(*a) Describe all statistical methods, including those used to control for confounding  **Text:**  “Regression models were fit to estimate the effects of individual and country-level factors on vaccine immunogenicity outcomes while controlling for potential confounders. Mixed effect logistic regression and mixed effect linear regression models were used to analyze the anti-rotavirus IgA seroconversion (dichotomous) and anti-rotavirus IgA antibody titer (continuous, log transformed) outcomes, respectively.”  “The modeling strategy for both outcomes began with variable specification, incorporating all individual-level characteristics as explanatory variables and controlling for potential country-level covariates selected based on variable distributions and existing literature.”  “Random effects were employed using a random intercept for each trial to account for potentially unmeasured differences between trial protocols or environments. The initial model included all explanatory variables of interest and incorporated interaction terms between each main effect and child mortality stratum. The model was applied to combined data from all child mortality strata and backwards elimination was then conducted using an α = 0.10 cut-off for variable inclusion, maintaining a hierarchically well-formulated model throughout.”  “Backwards elimination was subsequently performed after each variable was modified. Where initial models were prohibitively large and issues with model convergence were encountered, the most informative end models resulting from previous backwards elimination procedures were used as the starting point for investigation of refined measures. After all variables were explored, the most parsimonious model with all relevant variables and covariates was selected as the final model using Akaike information criterion (AIC) criteria (lower AIC indicating a better model) and assessed for multicollinearity using variance inflation factors (VIFs).” | Methods, 5^th^ paragraph  Methods, 7^th^ paragraph  Methods, 7^th^ paragraph  Methods, 8^th^ paragraph |
|  |  | (*b*) Describe any methods used to examine subgroups and interactions  **Text:**  “The initial model included all explanatory variables of interest and incorporated interaction terms between each main effect and child mortality stratum.”  “We examined whether the relationships identified using the full, combined dataset remained consistent within each mortality stratum; this would indicate that use of the combined dataset captured differences beyond those driven by child mortality stratum alone. To this end, a sensitivity analysis was conducted by fitting the final seroconversion model separately to data stratified by high and low child mortality settings, as well as moderately low and very low child mortality settings.”  “In addition, the final seroconversion model was tested using a subset of the data comprised of infants who were confirmed to be seronegative prior to vaccination. This sub-analysis was conducted to confirm that results produced from analysis of the full seroconversion dataset were consistent with results using infants with both pre- and post-vaccine serology. If consistent, this would suggest that inclusion of infants without pre-vaccine serology data was not causing substantial bias in our estimates.” | Methods, 7^th^ paragraph  Methods, 2^nd^ to last paragraph  Methods, 2^nd^ to last paragraph |
|  |  | (*c*) Explain how missing data were addressed  **Text:**  “Because our analysis was restricted to infants who participated in the trials according to protocol, the only instances of missing data were related to LAZ; height was not included in the protocols of a few studies and infants without these measurements were excluded in the models where LAZ was used as a predictor (seroconversion models: n = 691 (9%), IgA titer models: n = 525 (10%).” | Methods, 7^th^ paragraph |
|  |  | (*d*) If applicable, describe analytical methods taking account of sampling strategy  **Text:**  “Random effects were employed using a random intercept for each trial to account for potentially unmeasured differences between trial protocols or environments.” | Methods, 7^th^ paragraph |
|  |  | (*e*) Describe any sensitivity analyses  **Text:**  “We examined whether the relationships identified using the full, combined dataset remained consistent within each mortality stratum; this would indicate that use of the combined dataset captured differences beyond those driven by child mortality stratum alone. To this end, a sensitivity analysis was conducted by fitting the final seroconversion model separately to data stratified by high and low child mortality settings, as well as moderately low and very low child mortality settings.”  “In addition, the final seroconversion model was tested using a subset of the data comprised of infants who were confirmed to be seronegative prior to vaccination. This sub-analysis was conducted to confirm that results produced from analysis of the full seroconversion dataset were consistent with results using infants with both pre- and post-vaccine serology. If consistent, this would suggest that inclusion of infants without pre-vaccine serology data was not causing substantial bias in our estimates.” | Methods, 2^nd^ to last paragraph  Methods, 2^nd^ to last paragraph |
| Results | | | |
| Participants | 13* | (a) Report numbers of individuals at each stage of study—eg numbers potentially eligible, examined for eligibility, confirmed eligible, included in the study, completing follow-up, and analysed  **Text:**  “Data were available for analysis on 7,298 infants whose post-vaccine rotavirus serology data were collected 4-12 weeks after receipt of the final vaccine dose (87.8% of the rotavirus immunogenicity cohort). Of these infants, 39.0% (n = 2,849) were from high child mortality settings. A total of 18 infants (3 from high child mortality settings) were excluded from analysis due to pre-vaccine serology data indicating these children had prior rotavirus infection (i.e. anti-rotavirus IgA titer of ≥20 U/mL prior to the first rotavirus vaccine dose). All other infants had either confirmed seronegative status (4,988, 68.5%) or did not have serology data available (2,292, 31.5%) and were assumed to be seronegative based on trial protocol. Data on 7,280 and 5,161 infants were included in the anti-rotavirus IgA seroconversion and anti-rotavirus IgA titer modeling, respectively.” | Results, 1^st^ paragraph |
|  |  | (b) Give reasons for non-participation at each stage  **Text:**  “Since the primary aim of the analysis was to examine factors associated with rotavirus vaccine immunogenicity, data were limited to trial participants who received RV1 (n = 53,292, placebo groups excluded). Data were further restricted to infants whose trial participation was completed according to protocol (classified by GSK) and who participated in the rotavirus immunogenicity sub-studies of the trials (n = 8,309). Lastly, infants who had serum sample collection approximately 4-12 weeks from receipt of his/her last rotavirus vaccine dose were included (n = 7,298).”  “A total of 18 infants (3 from high child mortality settings) were excluded from analysis due to pre-vaccine serology data indicating these children had prior rotavirus infection (i.e. anti-rotavirus IgA titer of ≥20 U/mL prior to the first rotavirus vaccine dose)… Data on 7,280 and 5,161 infants were included in the anti-rotavirus IgA seroconversion and anti-rotavirus IgA titer modeling, respectively.” | Methods, 2^nd^ paragraph  Results, 1^st^ paragraph |
|  |  | (c) Consider use of a flow diagram  **Text:**  N/A | N/A |
| Descriptive data | 14* | (a) Give characteristics of study participants (eg demographic, clinical, social) and information on exposures and potential confounders  **Text:**  “All infants received either two or three doses of rotavirus vaccine (Table 3); nearly all infants in low child mortality settings received two doses (99%) while nearly half of infants in high child mortality settings received a third dose because of study protocols (45%). In low child mortality settings, one-quarter of infants received a reduced concentration of the vaccine (viral suspension <10^6.0^ CCID_50_), whereas all infants from high child mortality settings received a “standard” concentration (viral suspension ≥10^6.0^ CCID_50_).”  Table 3  “Individual-level characteristics frequently differed by setting (Table 3). Over 20% of infants in high child mortality settings were stunted or severely stunted while less than 10% were in low child mortality settings (p <0.001). All infants in high child mortality settings received OPV and over 90% of infants who received OPV received it concomitantly with both their first and second doses of rotavirus vaccine. In contrast, nearly half of infants in low child mortality settings did not receive any OPV (as IPV was more commonly administered in these settings). The percentage of children who seroconverted among the OPV categories ranged from 50% among those who received OPV concomitantly with only their first rotavirus vaccine dose to 83% among infants who did not receive OPV (Table 4). The median age at receipt of the first rotavirus dose was 9 weeks (IQR = 7, 11) and infants were a median of 22 weeks of age (IQR = 21, 26) when their post-vaccine serology sample was collected. The time from receipt of the last rotavirus dose to serology sample was slightly longer for infants in low child mortality settings (Table 3, median of 8 weeks, IQR = 5, 9) compared to infants in high child mortality settings (5 weeks, IQR = 5, 5, p <0.001).” | Results, 2^nd^ paragraph  Table 3  Results, 4^th^ paragraph |
|  |  | (b) Indicate number of participants with missing data for each variable of interest  **Text:**  Table 3  Table 4 and 5 | Table 3  Table 4 & 5 |
| Outcome data | 15* | Report numbers of outcome events or summary measures  **Text:**  “A majority of infants (70%) were seropositive after vaccination (Table 3) with a higher proportion of infants in low child mortality settings seroconverting (77%) compared to infants in high child mortality settings (62%, p <0.001). Anti-rotavirus IgA seroconversion ranged from 58% in India to over 90% in Hong Kong, Italy and Chile (Fig 2a). A similar pattern was found with post-vaccine anti-rotavirus IgA titer where infants in low child mortality settings had geometric mean titers (240, SD = 4) higher than that of infants in high child mortality settings (199, SD = 4, p <0.001). Anti-rotavirus IgA titer ranged from a median of 34 U/mL in India to 443 U/mL in Japan (Fig 2b).”  Table 4 and 5  Figure 2 | Results, 5^th^ paragraph  Table 4 & 5  Figure 2 |
| Main results | 16 | *(*a) Give unadjusted estimates and, if applicable, confounder-adjusted estimates and their precision (eg, 95% confidence interval). Make clear which confounders were adjusted for and why they were included  **Text:**  Table 4 and 5  “No association with seroconversion was identified for vaccine concentration, sex, age at first rotavirus vaccine dose, time from last rotavirus vaccine dose to post-vaccine serology sample, and age at post-vaccine serology sample in unadjusted analyses (Tables 4a and 4b). In the unadjusted analysis, infants who received three rotavirus vaccine doses were less likely to seroconvert compared to those who received two doses (unadjusted OR = 0.57, 95% CI = 0.50, 0.64, p < 0.001) as nearly three-quarters (73.6%) of children who received three doses were from high child mortality settings where seroconversion was less common. Those who received OPV concomitantly with both their first and second doses of rotavirus vaccine had reduced odds of seroconversion compared to those who did not receive OPV concomitantly (unadjusted OR = 0.58, 95% CI = 0.51, 0.66, p < 0.001). A similar relationship was observed for infants who received OPV concomitantly with only their first or second rotavirus dose, though the sample size for these two categories was very small. Child mortality status (OR = 0.48, 95% CI = 0.43, 0.53, p < 0.001) and under 5 mortality rate (OR = 0.69, 95% CI = 0.66, 0.72, p < 0.001) were negatively associated with seroconversion while GDP was positively associated with seroconversion (OR = 1.24, 95% CI = 1.20, 1.28, p < 0.001), as expected. The unadjusted associations between each variable and vaccine titer were generally similar to those observed for seroconversion.”  “Infants who received OPV concomitantly with both their first and second doses of rotavirus vaccine had 0.63 times the odds of seroconverting (OR = 0.63, 95% CI = 0.47, 0.84) compared to infants who received OPV but not concomitantly with either dose.”  Table 6  Table 7 | Table 4a & 4b  Results, 3^rd^ paragraph  Results, 6^th^ paragraph  Table 6  Table 7 |
|  |  | (*b*) Report category boundaries when continuous variables were categorized  **Text:**  N/A | N/A |
|  |  | (*c*) If relevant, consider translating estimates of relative risk into absolute risk for a meaningful time period  **Text:**  N/A | N/A |
| Other analyses | 17 | Report other analyses done—eg analyses of subgroups and interactions, and sensitivity analyses  **Text:**  “When the final anti-rotavirus IgA seroconversion model was applied to each mortality stratum separately (high, low, moderately low, and very low child mortality), similar results were observed (S2 Table). The negative relationship between OPV concomitant with both rotavirus doses 1 and 2 remained within each mortality setting, though with less precision of the parameter estimate. Similarly, when including only infants confirmed to be seronegative prior to rotavirus vaccination, results were consistent with the original analysis (S3 Table). Infants who received OPV concomitantly with rotavirus dose 1 and dose 2 were less likely to seroconvert than others (OR = 0.62, 95% CI = 0.46, 0.81, p = 0.001). The relationships between other variables and anti-rotavirus IgA seroconversion displayed only modest changes. Lastly, when the final model was fitted excluding infants who received OPV concomitantly with only their first or second rotavirus vaccine doses, similar results were observed (S4 Table).”  S1-S4 Tables | Results, 2^nd^ to last paragraph  S1-S4 Tables |
| Discussion | | | |
| Key results | 18 | Summarise key results with reference to study objectives  **Text:**  “We had the unique ability to pool individual-level data from 22 clinical trials and 33 countries/territories to create a dataset that enabled multilevel assessment of RV1 immunogenicity across settings. The resulting dataset was substantially larger than that of any individual trial or other related study [22]. Our findings suggest that OPV given concomitantly with rotavirus vaccine reduces anti-rotavirus IgA seroconversion even after two rotavirus vaccine doses. We did not find the same modifiable characteristics to be associated with post-vaccine anti-rotavirus IgA titers among infants who seroconverted, suggesting that such factors may predict whether an infant responds to rotavirus vaccination, but not the intensity of the response given seroconversion.” | Discussion, 1^st^ paragraph |
| Limitations | 19 | Discuss limitations of the study, taking into account sources of potential bias or imprecision. Discuss both direction and magnitude of any potential bias  **Text:**  “There are important challenges and limitations with this approach. First, while trial protocols were remarkably similar, trials themselves still differed by location, year, and population. We attempted to account for this variability by including a random effect for trial in our models. The required adherence to study protocol and stringent monitoring necessary for a clinical trial means that the results produced from these analyses may not perfectly reflect the findings that would have occurred under more routine, real-world conditions and should, therefore, be interpreted accordingly. Relatedly, the infants included in the trials were all healthy children, potentially limiting the generalizability of the results. Second, this is a secondary analysis of data previously collected for other primary purposes. We lacked data to control for genetic, maternal, socioeconomic and environmental factors that likely influence individual-level immune response to vaccination. To mitigate residual confounding from factors such as socioeconomic status or environment, a proxy measure (GDP) was included in the models. Third, of the 7,280 infants included in the analysis, 2,292 (31.5%) did not have pre-vaccine serology data. We addressed this limitation by conducting a sensitivity analysis in which the anti-rotavirus seroconversion model was applied to only infants confirmed to be seronegative prior to vaccination and we found the effect of OPV to be similar. Lastly, our analysis was limited to infants who received RV1 and it is possible that the findings and implications of this research may not be generalizable to the other three rotavirus vaccines prequalified by WHO or others currently in development.” | Discussion, 6^th^ paragraph |
| Interpretation | 20 | Give a cautious overall interpretation of results considering objectives, limitations, multiplicity of analyses, results from similar studies, and other relevant evidence  **Text:**  **“**These data provide robust evidence that infants who received OPV with both the first and second doses of RV1 were substantially less likely to seroconvert when compared to those not receiving OPV concomitantly This analysis bolsters existing evidence [32–36] that OPV may interfere with rotavirus seroconversion and suggests that OPV may interfere with seroconversion when given with both the first and second rotavirus vaccine doses. Early evidence indicated that the OPV-rotavirus interaction may be strongest for the first rotavirus dose with more recent evaluations suggesting lower seroconversion rates after completion of the full rotavirus vaccine course [32,33,35,37]. With this much larger and global dataset, our findings provide support for more recent data [32,35] that suggests a second rotavirus vaccine dose may not compensate for the reduced initial response. Applying our final seroconversion model to each mortality stratum individually and conducting a separate sensitivity analysis restricting data to only infants with confirmed pre-vaccine seronegative status provided support, as the direction of the relationship remained despite dropping a substantial portion of the study data. This finding is consistent with the hypothesis the OPV interferes with rotavirus vaccine response, and this effect is not explained by socioeconomic differences among the trial sites.”  “Comparing the adjusted OR for GDP and seroconversion developed via model selection (Table 5, OR = 1.11, 95% CI: 1.04-1.19, p = 0.001) to their crude association (OR = 1.24, 95% CI: 1.20, 1.28, p < 0.001) suggests that a substantial, though incomplete portion of the crude association was accounted for with our individual-level factors.”  “While we explored a number of potential factors, our findings highlight the importance of concomitant OPV administration and provide encouraging evidence to suggest OPV withdrawal could improve rotavirus vaccine performance.” | Discussion, 2^nd^ paragraph  Discussion, 4^th^ paragraph  Discussion, last paragraph |
| Generalisability | 21 | Discuss the generalisability (external validity) of the study results  **Text:**  “The required adherence to study protocol and stringent monitoring necessary for a clinical trial means that the results produced from these analyses may not perfectly reflect the findings that would have occurred under more routine, real-world conditions and should, therefore, be interpreted accordingly. Relatedly, the infants included in the trials were all healthy children, potentially limiting the generalizability of the results.”  “Lastly, our analysis was limited to infants who received RV1 and it is possible that the findings and implications of this research may not be generalizable to the other three rotavirus vaccines prequalified by WHO or others currently in development.” | Discussion, 6^th^ paragraph  Discussion, 6^th^ paragraph |
| Other information | | | |
| Funding | 22 | Give the source of funding and the role of the funders for the present study and, if applicable, for the original study on which the present article is based  **Text:**  “JMB received financial support to conduct this research from the Laney Graduate School of Emory University (<http://www.graduateschool.emory.edu>). BAL’s work was supported by NIH grants R01AI112970 and R01GM124280 (<https://grants.nih.gov/grants/oer.htm>) and the *Modeling the Impact of Rotavirus Vaccination in GAVI Countries* project supported by Imperial College London (<https://www.imperial.ac.uk>). The funders had no role in study design, data collection and analysis, decision to publish, or preparation of the manuscript.” | Funding disclosure statement |

*Give information separately for exposed and unexposed groups.

**Note:** An Explanation and Elaboration article discusses each checklist item and gives methodological background and published examples of transparent reporting. The STROBE checklist is best used in conjunction with this article (freely available on the Web sites of PLoS Medicine at http://www.plosmedicine.org/, Annals of Internal Medicine at http://www.annals.org/, and Epidemiology at http://www.epidem.com/). Information on the STROBE Initiative is available at www.strobe-statement.org.
