# Supplementary material for: Antirotavirus IgA seroconversion rates in children who receive concomitant oral poliovirus vaccine: A secondary, pooled analysis of Phase II and III trial data from 33 countries
Source: PLoS Med. 2019 Dec 30;16(12):e1003005. doi: 10.1371/journal.pmed.1003005 (PMC6936798; doi:10.1371/journal.pmed.1003005)
Supplement: S1 Text — Basic model formulas for mixed-effect logistic regression and mixed-effect linear regression of log-transformed data for antirotavirus IgA seroconversion (dichotomous) and antirotavirus IgA titer (continuous) outcomes, respectively. IgA, immunoglobulin A. (DOCX) [file pmed.1003005.s002.docx]

**S1 Text. Model equations**

Logistic model: Logit P(Y_ij_) = b_0j_ + β_0_ + β_a1…an_(H) + β_b1…bn_(C) + β_c1…cn_(H*C)

Y_ij_ represents anti-rotavirus IgA ≥20 U/mL for the *i*th infant, in the *j*th trial

b_0j_ represents a random intercept for each trial

β_0_ represents the intercept

β_a-c_ represent regression coefficients for host, country and interaction term, respectively

H represents a vector of host characteristics, a_1_ through a_n_

C represents a vector of country factors, b_1_ through b_n_

H*C represents a vector of interaction terms for host/country characteristics and child

mortality strata

Linear model: Ln(Y_ij_) = b_0j_ + β_0_ + β_a1…an_(H) + β_b1…bn_(C) + β_c1…cn_(H*C) + ε

Y_ij_ represents the anti-rotavirus IgA antibody titer for the *i*th infant, in the *j*th trial

b_0j_ represents a random intercept for each trial

β_0_ represents the intercept

β_a-c_ represent regression coefficients for host, country and interaction term, respectively

H represents a vector of host characteristics, a_1_ through a_n_

C represents a vector of country factors, b_1_ through b_n_

H*C represents a vector of interaction terms for host/country characteristics and child

mortality strata

ε represents error
