# Supplementary material for: Antirotavirus IgA seroconversion rates in children who receive concomitant oral poliovirus vaccine: A secondary, pooled analysis of Phase II and III trial data from 33 countries
Source: PLoS Med. 2019 Dec 30;16(12):e1003005. doi: 10.1371/journal.pmed.1003005 (PMC6936798; doi:10.1371/journal.pmed.1003005)
Supplement: S2 Text — (DOCX) [file pmed.1003005.s003.docx]

**S2 Text. Analysis plan**

Aim: Estimate the effects of individual and country-level factors on vaccine immunogenicity outcomes while controlling for potential confounders.

*Pre-specified analysis plan*

1. Explore data using univariate and bivariate analysis.
2. Select predictors and covariates to build initial model based on *data exploration and correlation analysis*.
3. Add interaction terms to model for each main effect with country’s child mortality status to assess effect modifications.
4. Begin backwards elimination starting with the interaction terms.
   1. Drop the most non-significant interaction term and re-run model.
   2. Repeat step 3 until all interaction terms are significant at the α= 0.10 level.
   3. Drop the most non-significant main effect term, maintaining any main effect that is included in an interaction term so model remains hierarchically well formulated. Re-run model.
   4. Repeat step 4 until all main effect terms are significant at the α= 0.10 level or cannot be dropped because they are included in an interaction term.
5. Refine models by including a modified version of the predictors in the model (e.g. more detailed OPV and age at post-vax serology squared).
   1. Repeat steps 3-5
6. Select final most parsimonious model with all relevant variables and covariates using Akaike information criterion (AIC) criteria (lower AIC indicating a better model).
7. Conduct sensitivity analysis by applying the final model from step 6 to each child mortality stratum.

*Analyses added post-hoc*

1. Assess final model for multicollinearity using variance inflation factors (VIFs).
2. Calculate population attributable fraction and attributable fraction among the exposed for variables of interest.

*Data exploration and correlation analysis*

1. Univariate analysis: examine distribution of variables for all countries combined and stratified by child mortality status
   1. Post-vaccine IgA titer
      1. Standard, unscaled IgA titer
      2. Ln(IgA titer)
   2. Seroconversion
   3. Time from last rotavirus dose to serology sample/outcome measure (weeks)
   4. Age at post-vaccine serology sample
   5. Age at first vaccine dose (weeks)
      1. Standard measure
      2. Age at first dose squared
   6. Age at each rotavirus vaccine dose (weeks)
   7. Number of rotavirus vaccine doses
   8. Vaccine concentration
   9. Sex
   10. Length-for-age z-score (LAZ), represents nutritional status
       1. Continuous
       2. Stratified as severe stunting, stunting, no stunting
   11. OPV
       1. Number of OPV doses
       2. OPV concomitant with rotavirus dose 1, dose 2, and both dose 1 and dose 2, neither dose
       3. OPV +/- 14 days of rotavirus doses
   12. Trial number
2. Bivariate analysis for all countries combined and stratified by child mortality status
   1. Predictors with one another
      1. Examine correlation table
   2. Each predictor with outcome measures (seroconversion/ln(IgA titer))
3. Select predictors and covariates for initial model based on univariate and bivariate analysis.

*Final modeling strategy*

1. Variable specification
   1. Individual-level characteristics in initial model
      1. Time from last rotavirus dose to serology sample/outcome measure (weeks)
      2. Number of rotavirus vaccine doses (2 vs. 3)
      3. Age at first vaccine dose (weeks)
      4. Vaccine concentration (standard vs. low)
      5. Sex (female vs. male)
      6. Length-for-age z-score (LAZ)
      7. Concomitant OPV
   2. Country-level covariates in initial model
      1. GDP (scaled)
      2. Under 5 mortality rate
      3. Under 5 mortality stratum (dichotomous).
   3. Random effects (random intercept) for each trial to account for potentially unmeasured differences between trial protocols or environments.
2. Models for seroconversion (dichotomous)
   1. Run backwards elimination to develop final model for data including all countries
   2. Select the most parsimonious model with all relevant variables and covariates using Akaike information criterion (AIC) criteria as the final model.
   3. Assess final model for multicollinearity using variance inflation factors (VIFs).
   4. Apply final model to data stratified by child mortality status (high, low, moderately low, very low).
   5. Apply final model to data restricted to infants with confirmed seronegative status prior to first rotavirus vaccine dose.
3. Models for ln(IgA) titer outcome (continuous) among those who seroconverted after vaccination
   1. Repeat steps 2a-2c for ln(IgA) titer outcome.
