# Supplementary material for: Antirotavirus IgA seroconversion rates in children who receive concomitant oral poliovirus vaccine: A secondary, pooled analysis of Phase II and III trial data from 33 countries
Source: PLoS Med. 2019 Dec 30;16(12):e1003005. doi: 10.1371/journal.pmed.1003005 (PMC6936798; doi:10.1371/journal.pmed.1003005)
Supplement: S1 Table — (DOCX) [file pmed.1003005.s004.docx]

| **Very Low**  **Child Mortality** | **Moderately Low**  **Child Mortality** | **High Child Mortality** |  |
| --- | --- | --- | --- |
| Canada  Czech Republic  Finland  France  Germany  Hong Kong  Italy  Japan  Korea, Republic of  Poland  Portugal  Singapore  Spain  Taiwan  United States | Argentina  Brazil  Chile  China  Colombia  Honduras  Mexico  Nicaragua  Panama  Peru  Philippines  Venezuela  Vietnam | Bangladesh  Dominican Republic  India  Malawi  South Africa | |
