# Supplementary material for: Antirotavirus IgA seroconversion rates in children who receive concomitant oral poliovirus vaccine: A secondary, pooled analysis of Phase II and III trial data from 33 countries
Source: PLoS Med. 2019 Dec 30;16(12):e1003005. doi: 10.1371/journal.pmed.1003005 (PMC6936798; doi:10.1371/journal.pmed.1003005)
Supplement: S2 Table — IgA, immunoglobulin A. (DOCX) [file pmed.1003005.s005.docx]

|  | **Child mortality level** | | | | | | | |
| --- | --- | --- | --- | --- | --- | --- | --- | --- |
|  | **High**  **(n = 2,806)** | | **Low**  **(n = 3,783)** | | **Moderately low**  **(n = 1,836)** | | **Very low**  **(n = 1,947)** | |
| **Individual or country-level factor** | **OR (95% CI)** | **p-value** | **OR (95% CI)** | **p-value** ^a^ | **OR (95% CI)** | **p-value** ^a^ | **OR (95% CI)** | **p-value** ^a^ |
| Time from last rotavirus dose to serology (per week) | 0.97 (0.83, 1.13) | 0.665 | 0.88 (0.84, 0.93) | <0.001 | 0.92 (0.85, 1.00) | 0.045 | 0.85 (0.79, 0.90) | <0.001 |
| Age at 1st rotavirus dose (weeks) | 1.02 (0.99, 1.05) | 0.210 | 1.13 (1.09, 1.18) | <0.001 | 1.14 (1.08, 1.21) | <0.001 | 1.13 (1.07, 1.20) | <0.001 |
| Vaccine concentration ≥10^6.0^ | 1.00 (ref) |  | 1.00 (ref) |  | 1.00 (ref) |  | 1.00 (ref) |  |
| Vaccine concentration <10^6.0 b^ | **--** |  | 0.62 (0.45, 0.83) | 0.002 | 0.49 (0.23, 1.07) | 0.074 | **--** |  |
| LAZ: stunted or severely stunted | 1.00 (ref) |  | 1.00 (ref) |  | 1.00 (ref) |  | 1.00 (ref) |  |
| LAZ: not stunted/severely stunted | 0.84 (0.68, 1.03) | 0.093 | 1.24 (0.93, 1.65) | 0.138 | 1.27 (0.88, 1.83) | 0.198 | 1.19 (0.76, 1.85) | 0.450 |
| OPV neither concomitant w/ rotavirus dose 1 nor 2 | 1.00 (ref) |  | 1.00 (ref) |  | 1.00 (ref) |  | 1.00 (ref) |  |
| OPV concomitant w/ rotavirus dose 1 & 2 | 0.71 (0.40, 1.26) | 0.246 | 0.61 (0.42, 0.89) | 0.011 | 0.63 (0.42, 0.93) | 0.020 | -- |  |
| OPV concomitant w/ rotavirus dose 1 only | 0.49 (0.13, 1.83) | 0.287 | 0.17 (0.02, 1.95) | 0.156 | 0.18 (0.02, 2.01) | 0.163 | -- |  |
| OPV concomitant w/ rotavirus dose 2 only | 0.40 (0.09, 1.83) | 0.239 | 0.91 (0.30, 4.44) | 0.862 | 0.95 (0.31, 2.97) | 0.935 | -- | 0.479 |
| No OPV received ^c^ | -- |  | 0.78 (0.40, 1.53) | 0.474 | -- |  | 2.40 (0.21, 26.89) |  |
| Log(GDP) | 1.10 (1.02, 1.17) | 0.009 | 1.28 (1.02, 1.60) | 0.035 | 1.37 (0.97, 1.94) | 0.076 | 1.17 (0.85, 1.63) | 0.338 |

^a^ Determined by Wald Test.

^b^ No children in high child mortality settings or very low child mortality settings received low concentration vaccines.

^c^ All children in high and moderately low child mortality setting received at least one dose of OPV.

OR, odds ratio; CI, confidence interval; LAZ, length-for-age z-score; OPV, oral poliovirus vaccine, GDP, gross domestic product; ref, reference group.
