# Supplementary material for: Antirotavirus IgA seroconversion rates in children who receive concomitant oral poliovirus vaccine: A secondary, pooled analysis of Phase II and III trial data from 33 countries
Source: PLoS Med. 2019 Dec 30;16(12):e1003005. doi: 10.1371/journal.pmed.1003005 (PMC6936798; doi:10.1371/journal.pmed.1003005)
Supplement: S3 Table — IgA, immunoglobulin A. (DOCX) [file pmed.1003005.s006.docx]

| **Individual or country-level factor** | **OR (95% CI)** | **p-value** ^a^ |
| --- | --- | --- |
| Time from last rotavirus dose to serology (per week) | 0.88 (0.84, 0.93) | <0.001 |
| Vaccine concentration ≥10^6.0^ | 1.00 (ref) |  |
| Vaccine concentration <10^6.0^ | 0.65 (0.48, 0.87) | 0.003 |
| OPV neither concomitant w/ rotavirus dose 1 nor 2 | 1.00 (ref) |  |
| OPV concomitant w/ rotavirus dose 1 & 2 | 0.62 (0.46, 0.81) | 0.001 |
| OPV concomitant w/ rotavirus dose 1 only | 0.36 (0.05, 2.58) | 0.309 |
| OPV concomitant w/ rotavirus dose 2 only | 0.90 (0.30, 2.70) | 0.855 |
| No OPV received | 0.92 (0.56, 1.50) | 0.733 |
| Log(GDP) | 1.20 (1.05, 1.38) | 0.008 |
| Age at 1st rotavirus dose (weeks) | 1.13 (1.08, 1.17) | <0.001 |
| Age at 1^st^ rotavirus dose (weeks)*Child mortality setting | 0.89 (0.84, 0.95) | <0.001 |
| LAZ: stunted or severely stunted | 1.00 (ref) |  |
| LAZ: not stunted/severely stunted | 1.22 (0.92, 1.62) | 0.173 |
| LAZ: not stunted*Child mortality setting | 0.64 (0.40, 1.04) | 0.071 |
| Child mortality- Low child mortality settings | 1.00 (ref) |  |
| Child mortality- High child mortality settings | 1.80 (0.77, 4.22) | 0.178 |

^a^ Determined by Wald Test; IgA, immunoglobulin A; OR, odds ratio; CI, confidence interval; LAZ, length-for-age z-score; OPV, oral poliovirus vaccine, GDP, gross domestic product; ref, reference group.
