# Supplementary material for: Antirotavirus IgA seroconversion rates in children who receive concomitant oral poliovirus vaccine: A secondary, pooled analysis of Phase II and III trial data from 33 countries
Source: PLoS Med. 2019 Dec 30;16(12):e1003005. doi: 10.1371/journal.pmed.1003005 (PMC6936798; doi:10.1371/journal.pmed.1003005)
Supplement: S4 Table — IgA, immunoglobulin A; OPV, oral poliovirus vaccine. (DOCX) [file pmed.1003005.s007.docx]

| **Individual or country-level factor** | **OR (95% CI)** | **p-value** ^a^ |
| --- | --- | --- |
| Time from last rotavirus dose to serology (per week) | 0.90 (0.86, 0.94) | <0.001 |
| Vaccine concentration ≥10^6.0^ | 1.00 (ref) |  |
| Vaccine concentration <10^6.0^ | 0.65 (0.49, 0.87) | 0.003 |
| OPV neither concomitant w/ rotavirus dose 1 nor 2 | 1.00 (ref) |  |
| OPV concomitant w/ rotavirus dose 1 & 2 | 0.63 (0.48, 0.85) | 0.002 |
| No OPV received | 1.14 (0.76, 1.70) | 0.524 |
| Log(GDP) | 1.11 (1.04, 1.18) | 0.002 |
| Age at 1^st^ rotavirus dose (weeks) | 1.13 (1.08, 1.17) | <0.001 |
| Age at 1^st^ rotavirus dose (weeks)*Child mortality setting | 0.90 (0.86, 0.95) | <0.001 |
| LAZ: stunted or severely stunted | 1.00 (ref) |  |
| LAZ: not stunted/severely stunted | 1.24 (0.93, 1.65) | 0.139 |
| LAZ: stunted or severely stunted*Child mortality setting | 0.68 (0.48, 0.96) | 0.030 |
| Child mortality setting- low | 1.00 (ref) |  |
| Child mortality setting- high | 1.62 (0.83, 3.14) | 0.154 |

^a^ Determined by Wald Test; OR, odds ratio; CI, confidence interval; LAZ, length-for-age z-score; OPV, oral poliovirus vaccine; GDP, gross domestic product; ref, reference group.
